# Draft genomes of two *Escherichia marmotae* strains isolated from animal feces from a grain field in Germany

**DOI:** 10.1128/mra.01365-25

**Published:** 2026-03-27

**Authors:** Michaela Projahn, Steffen Hermann, Ulrike Binsker, Christian Menge, Elisabeth Schuh

**Affiliations:** 1 Department of Biological Safety, National Reference Laboratory for Escherichia coli including VTEC, German Federal Institute for Risk Assessment 27652, Berlin, Germany; 2 Friedrich-Loeffler-Institute, Institute of Animal Nutrition, Braunschweig, Germany; 3 Department of Biological Safety, National Reference Laboratory for Antimicrobial Resistance, German Federal Institute for Risk Assessment 27652, Berlin, Germany; 4 Friedrich-Loeffler-Institute, Institute of Molecular Pathogenesis, Jena, Germany; Wellesley College, Wellesley, Massachusetts, USA

**Keywords:** *Escherichia marmotae*, genome, wild animal, DNA sequencing

## Abstract

The species *Escherichia marmotae* was newly assigned in 2015. Since then, few isolates have been identified from different animal species or humans. In addition, antibiotic resistance has been detected in this species. We present the draft genomes of two *E. marmotae* strains from animal feces collected from a grain field.

## ANNOUNCEMENT

*Escherichia marmotae* was first isolated from *Marmota himalayana* feces in 2015 (1). Further *E. marmotae* strains were isolated from various animals like rats, mice, or cattle (2–4), as well as environmental sources (5). In addition, human isolates were obtained from clinical cases with mild to severe symptoms (6–9) that indicate *E. marmotae* as a potential zoonotic pathogen in the One Health context. Genomic characterization of some strains revealed the appearance of antimicrobial resistance genes in this species (4, 7).

In our study, we analyzed feces from wild animals collected from a wheat grain field in Germany to identify possible contamination sources for the grain and the subsequent flour processing. Feces samples were collected separately into 50 mL Falcon tubes in February 2025 before the field was fertilized with manure and were stored at 4°C until further processing. Feces samples (1 g) were incubated in 9 mL buffered peptone water in a rotation incubator at 37°C and 175 rpm. An aliquot of 10 µL of the overnight cultures was streaked on MacConkey agar (Merck, Darmstadt, Germany) supplemented with 100 µg/mL ampicillin using an inoculation loop and incubated at 37°C for 24 h. Single colonies were isolated, and the species affiliation *Escherichia marmotae* was identified for two colonies from two different fecal samples (presumably wild boar based on the feces morphology) using matrix-assisted laser desorption/ionization time-of-flight mass spectrometry (MALDI-TOF MS) (Bruker Daltonics, Bremen, Germany). Antimicrobial susceptibility testing was performed by broth microdilution using the commercial EUVSEC3 microplates (Sensititre, Thermo Fisher Scientific), with the antimicrobial substances listed in Table 1 and concentration ranges and epidemiological cut-off values according to the Commission Implementing Decision 2020/1729/EU (10) and the European Committee on Antimicrobial Susceptibility Testing (EUCAST) (11). Genomic DNA was extracted using the PureLink Genomic DNA Mini Kit (Thermo Fisher Scientific, USA). Libraries were prepared with the Illumina DNA Prep (M) Tagmentation Kit (Illumina, San Diego, CA, USA) and sequenced on a NextSeq500 benchtop sequencer using the NextSeq 500/550 midoutput kit v2.5 (300-cycle, 2 × 149 bp paired-end). Raw reads were trimmed and *de novo* assembled using the AQUAMIS pipeline v1.4.2 (https://gitlab.com/bfr_bioinformatics/AQUAMIS/), which implements fastp v0.23.2 for trimming (12) and shovill v1.1.0 (https://github. com/tseemann/shovill, based on Spades) for assembly. The AQUAMIS pipeline includes quast v5.2.0 for assembly quality control (https://github. com/ablab/quast). Strain characterization was performed with the BakCharak pipeline v3.1.6 (https://gitlab.com/bfr_bioinformatics/bakcharak), which includes multi-locus sequence typing (MLST) (https://github.com/tseemann/mlst) and antimicrobial resistance (AMR) typing based on the NCBI AMRFinder (13). The DNA isolation and sequencing kits were used according to the manufacturer’s instructions. Default parameters were used for all software tools.

**TABLE 1:** Results from the genome sequencing and strain characteristics of the two *E. marmotae* isolates^*a*^

Characteristic	BfR-EC-20803	BfR-EC-20804
Sample no.	25-EC00288	25-EC00289
Sequence ID	25-EC00288-3	25-EC00289-2
BioSample accession no.	SAMN51305455	SAMN51305456
SRA accession no.	SRR35386461	SRR35386460
GenBank no.	JBSGOV000000000	JBSGOW000000000
No. of reads	2,487,212	2,722,057
No. of contigs	101	109
Q30 base fraction	0.870	0.869
Coverage depth	76.8	85.5
N50 (bp)	153,262	161,021
Total genome length (bp)	4,712,503	4,624,491
GC content (%)	50.29	50.38
MLST Warwick	ST5600	ST7630
MIC values		
AK (4–128 mg/L)	≤4	≤4
AMP (1–32 mg/L)	2	4
AZI (2–64 mg/L)	≤2	≤2
FOT (0.25–4 mg/L)	≤0.25	≤0.25
TAZ (0.25–8 mg/L)	0.5	0.5
CHL (8–64 mg/L)	≤8	≤8
CIP (0.015–8 mg/L)	≤0.015	≤0.015
COL (1–16 mg/L)	≤1	≤1
GEN (0.5–16 mg/L)	1	1
MERO (0.03–16 mg/L)	≤0.03	≤0.03
NAL (4–64 mg/L)	≤4	≤4
SMX (8–512 mg/L)	≤8	≤8
TET (2–32 mg/L)	≤2	≤2
TGC (0.25–8 mg/L)	≤0.25	≤0.25
TMP (0.25–16 mg/L)	≤0.25	≤0.25

^*a*^ The following antimicrobial substances were tested for the strains: amikacin (AK), ampicillin (AMP), azithromycin (AZI), cefotaxime (FOT), ceftazidime (TAZ), chloramphenicol (CHL), ciproﬂoxacin (CIP), colistin (COL), gentamicin (GEN), meropenem (MERO), nalidixic acid (NAL), sulfamethoxazole (SMX), tetracycline (TET), tigecycline (TGC), and trimethoprim (TMP).

**Fig 1:**
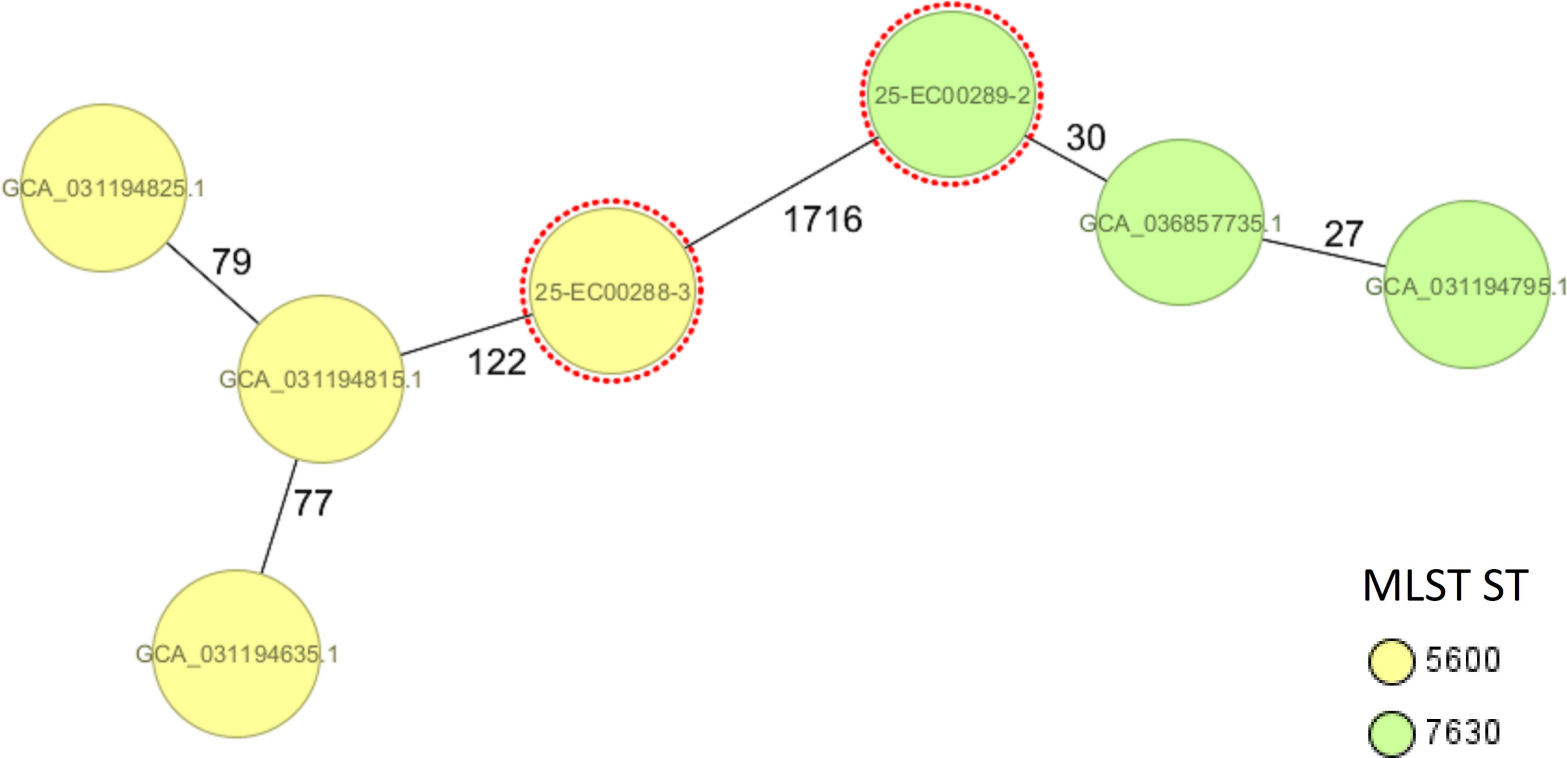
Minimum spanning tree (MST) of the newly identified *E. marmotae* strains (red circles) in feces collected from a wheat grain field compared to *E. marmotae* strains from the study of reference 14. The MST was calculated using the Ridom SeqSphere software v10.5.0 (2025-03) with the Enterobase cgMLST scheme comprising 2,513 loci. Colors highlight the respective MLST ST; red circles represent the two newly identified strains from this study.

Strains were assigned to MLST ST5600 and ST7630 (Table 1), respectively, which were previously identified in a larger study in Germany comprising *E. marmotae* strains isolated from animals and food between 2016 and 2020 (14). Strains of the respective MLST ST from that study were compared to the two strains using the core-genome multi-locus sequence typing (cgMLST) tool for *E. coli* implemented in the Ridom SeqSphere software v10.5.0 (2025-03) (Fig. 1), showing a closer relationship in the ST7630 strains than in the ST5600 strains. Antimicrobial resistance testing revealed that both strains were susceptible to all tested substances, supporting the genomic data, as no genetic antimicrobial resistance determinants were identified. Our findings underline that wild animals are a natural host for *E. marmotae* strains. However, the impact of *E. marmotae* strains in feces posing as a contamination source for grain and subsequently flour processing, possibly resulting in human infections, needs to be further investigated.

## Data Availability

All data are encompassed under BioProject accession number PRJNA1328070. Sequencing raw reads were deposited in the NCBI Sequence Read Archive (SRA) under accession numbers SRR35386461 and SRR35386460. Assembled genome data were deposited under GenBank numbers JBSGOV000000000 and JBSGOW000000000. The annotation of the draft genome files was added by the NCBI Prokaryotic Genome Annotation Pipeline (PGAP Version 6.10, 31 March 2025, https://www.ncbi.nlm.nih.gov/refseq/annotation_prok/) during upload.
